# Effect of Process Parameters on Nano-Microparticle Formation During Supercritical Antisolvent Process Using Mixed Solvent: Application for Enhanced Dissolution and Oral Bioavailability of Telmisartan Through Particle-Size Control Based on Experimental Design

**DOI:** 10.3390/pharmaceutics16121508

**Published:** 2024-11-24

**Authors:** Eun-Sol Ha, Heejun Park, Ji-Su Jeong, Seon-Kwang Lee, Hui-Taek Kang, In-hwan Baek, Min-Soo Kim

**Affiliations:** 1 College of Pharmacy and Research Institute for Drug Development, Pusan National University, 63 Busandaehak-ro, Geumjeong-gu, Busan 46241, Republic of Korea; edel@pusan.ac.kr (E.-S.H.); sui15@pusan.ac.kr (J.-S.J.); lsk7079@pusan.ac.kr (S.-K.L.); gms1406@pusan.ac.kr (H.-T.K.); 2 College of Pharmacy, Duksung Women’s University, 33, Samyangro 144-gil, Dobong-gu, Seoul 01369, Republic of Korea; heejunpark@duksung.ac.kr; 3 College of Pharmacy, Kyungsung University, 309, Suyeong-ro, Nam-gu, Busan 48434, Republic of Korea; baek@ks.ac.kr

**Keywords:** supercritical antisolvent process, nano-microparticles, bioavailability, telmisartan

## Abstract

**Background/Objectives:** This study investigates the impact of supercritical antisolvent (SAS) process parameters on the particle formation of telmisartan, a poorly water-soluble drug. **Methods:** A fractional factorial design was employed to examine the influence of the SAS process parameters, including solvent ratio, drug solution concentration, temperature, pressure, injection rate of drug solution, and CO₂ flow rate, on particle formation. Solid-state characterizations of the SAS process particles using XRD and FT-IR confirmed their amorphous nature. The effect of particle size on the kinetic solubility, dissolution, and oral bioavailability of telmisartan was also assessed. **Results:** Using a mixture of dichloromethane and methanol, telmisartan amorphous nano-microparticles with sizes between 200 and 2000 nm were produced. The key parameters, particularly drug solution concentration and temperature, significantly affected the particle size. Interestingly, the ratio of the solvent mixture also had a significant effect on the particle morphology. Further experiments were performed to determine the conditions for preparing telmisartan amorphous nano-microparticles with various sizes by controlling the solvent mixture ratio and the concentration of the drug solution. It was revealed that a reduction in the amorphous particle size enhanced both the kinetic solubility and dissolution rates, leading to a significantly increased in vivo oral bioavailability in rats compared to unprocessed telmisartan. **Conclusions:** These findings suggest that SAS processing, utilizing adjustments of process parameters, offers an effective strategy for enhancing the bioavailability of poorly soluble drugs by generating amorphous spherical nano-microparticles with optimized particle size.

## 1. Introduction

In the pharmaceutical industry, supercritical fluid (SCF) technology is a useful method for the production of micro- and nano-particles. SCFs are substances that exist at a temperature and pressure higher than their critical temperature and critical pressure, and have both gas and liquid properties. Carbon dioxide (CO_2_), which is most commonly used in SCF technology, is non-toxic, non-flammable, and has a low critical point of *T*_c_ = 304.1 K and *P*_c_ = 7.38 MPa; hence, it can be applied to formulation studies of heat-unstable components [1,2,3].

Among the various SCF technologies, the supercritical anti-solvent (SAS) process uses the SCF as an anti-solvent. In the general SAS process, an organic solvent in which the solute is completely dissolved is sprayed through a nozzle into a high-pressure vessel containing the SCF and is mixed with the fluid. The SCF dissolves in the solvent and the solution expands, reducing the solvating power of the solvent. Subsequently, supersaturation and particle precipitation result in the generation of new particles. Particles with various properties (size, size distribution, etc.) can be prepared by changing the process parameters of the SAS process [4,5,6]. Many studies have applied the SAS process using supercritical (SC) CO_2_ to prepare particles containing active pharmaceutical ingredients, finding that these techniques effectively enhance the solubility and oral bioavailability of drugs with limited water solubility. In addition, an advantage of the SAS process is that it produces solid pure drug nano-microparticles without using surface stabilizers or dispersants [4,5]. In this process, a drug solution is first prepared by dissolving the drug in an organic solvent. Yield-wise, it is advantageous to perform the SAS process by selecting a solvent with good solubility in the drug solution. For this reason, most studies on producing solid drug nanoparticles using the SAS process have mainly focused on drug solutions in a mono-organic solvent system where drug solubility can be sufficiently secured [7]. Meanwhile, research on the SAS process for drug solutions using mixed solvent systems is lacking. As the solvents that can be used in pharmaceutical processes are very limited, research on securing drug solubility through the use of mixed solvents and utilizing them in nano-microparticle formation is required to expand opportunities for further drug nano-microparticle development. In particular, a few studies prepared solid particles composed of only excipients using SAS with a solvent mixture [7,8]. It has been reported that the morphology and size of particles can be controlled by altering the composition of the solvent mixtures. However, these studies did not attempt in-depth research using systematic experimental design-based strategies, and to the best of our knowledge, no attempts were made to improve the pharmaceutical properties of drugs.

Telmisartan is a drug effective for treating hypertension, acting through the mechanism of an angiotensin receptor blockade owing to its high affinity for angiotensin II type 1 receptors [4,9,10]. However, telmisartan, a Biopharmaceutics Classification System (BCS) class II drug, faces issues of low solubility, slow dissolution rate, and low oral bioavailability owing to poor water solubility. To address these challenges, numerous studies have investigated various pharmaceutical technologies such as amorphous solid dispersions [11,12], solid nano-microparticles [13,14,15], water-soluble complex formation [16], co-crystals [10], and several lipid-based formulations [17,18,19]. Most of these studies have attempted to add stabilizers such as surfactants or approach the formulation aspect, and few have attempted to improve the pharmaceutical properties of a pure drug without inactive ingredients. Telmisartan, which has relatively low solubility in a single organic solvent, is difficult to apply to the SAS process because its supersaturation is not easy to control. However, in our previous study, it was revealed that telmisartan has a higher solubility in solvent mixtures with dichloromethane and methanol than in mono-solvent systems [9]; therefore, this solvent mixture system can be applied to the SAS process owing to its sufficient solvation power. Thus, telmisartan was selected as the model drug for this study.

In this study, the effect of SAS process parameters, focusing on the solvent ratio of dichloromethane in a mixture with methanol, on particle formation was investigated in combination with the drug solution concentration, temperature, pressure, injection rate, and CO_2_ flow rate, using fractional factorial design (FFD) and full factorial design (FD). The solid-state properties of telmisartan particles were characterized using dynamic light scattering (DLS), scanning electron microscopy (SEM), X-ray powder diffraction (XRD), and Fourier-transform infrared (FT-IR) spectroscopy. The effect of particle size on the kinetic solubility, dissolution, and oral bioavailability of telmisartan was also assessed.

## 2. Materials and Methods

### 2.1. Materials

Telmisartan (C_33_H_30_N_4_O_2_; Form A) was obtained from Dong-A ST Co., Ltd. (Seoul, Republic of Korea). Carbon dioxide (CO_2_, 99.9%) was supplied by Hana Gas Co. Ltd. (Gimhae, Republic of Korea). Raloxifene was purchased from Sigma-Aldrich Co. (St. Louis, MO, USA). High-performance liquid chromatography (HPLC)-grade dichloromethane and methanol were purchased from Honeywell Burdick and Jackson (Muskegon, MI, USA). All other chemicals were of extra pure grade.

### 2.2. SAS Process

**Figure 1:**
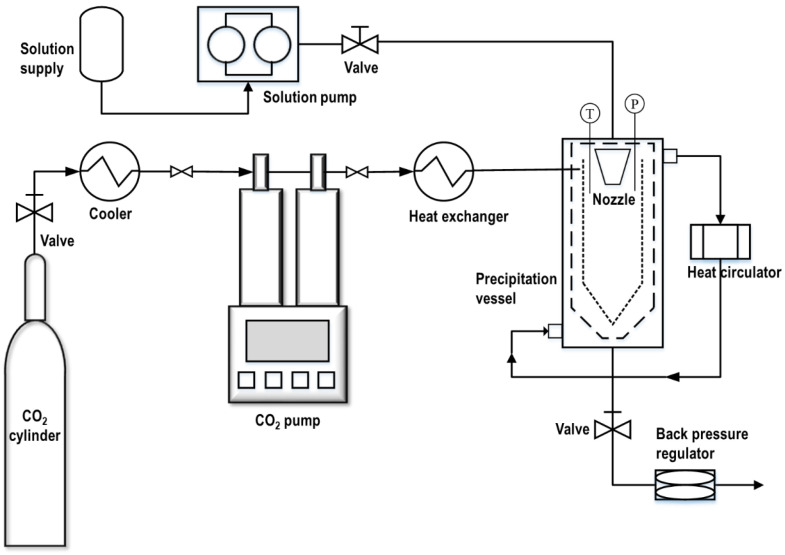
Schematic diagram of the SAS process.

A scheme of the SAS experimental equipment is shown in Figure 1. Using a low-temperature bath circulator (RW-0525G; Jeiotech Co., Ltd., Daejeon, Republic of Korea) set to −15 °C, the liquefied CO_2_ was transferred to a reactor (at approximately 100 mL) through the external line of a two way-fluid nozzle (inner diameter: 100 μm) using a cylinder syringe pump (Model 260D; Teledyne Technologies Inc., Thousand Oaks, CA, USA) and a heat exchanger. CO_2_ was continuously delivered at a constant rate using the cylinder syringe pump until the desired pressure was obtained, and the temperature of the reactor was maintained using a circulating water bath (CW-10G; Jeiotech Co., Ltd., Daejeon, Republic of Korea). Once the system reached an equilibrium, the drug solution was simultaneously sprayed at a constant rate through the internal line of a two way-fluid nozzle using an HPLC liquid pump (LS-Class pump; Teledyne Technologies Inc., Thousand Oaks, CA, USA), where particles were generated by rapid extraction of the used solvents with SC-CO_2_. Upon completion of the drug solution injection, CO_2_ was additionally pumped at the same rate to remove the residual solvent that could dissolve the prepared particles. The CO_2_ flow was then stopped, and the system was slowly depressurized to atmospheric pressure using a back-pressure regulator (26-1762-24; Tescom, Pflugerville, TX, USA). Finally, the precipitated particles were collected from the internal container basket located at the bottom of the reactor.

### 2.3. Evaluation of the Effects of SAS Process Parameters on Particle Formation Using Experimental Design

In the SAS process, the screening study was performed using an FFD to investigate the effects of various variables on particle size (Table 1). The independent variables were the solvent ratio of dichloromethane in mixture with methanol (X_1_), drug solution concentration (X_2_), temperature (X_3_), pressure (X_4_), drug solution injection rate (X_5_), and CO_2_ flow rate (X_6_), and the dependent variables were the particle size (Y_1_) and crystal form (Y_2_). The levels of the independent variables were selected based on the results of preliminary studies and reported values in the literature [4,9]. A total of 19 experimental points, which were run in random order, consisted of two levels (low and high) of six factors and three replications of a center point. The experimental design was generated and evaluated using the Design Expert 11.0 software (Stat-Ease, Minneapolis, MN, USA). Telmisartan’s solubility in commonly used organic solvents for the SAS process, namely methanol, ethanol, 2-propanol, acetonitrile, acetone, dichloromethane, and tetrahydrofuran, is below 10 mg/mL, while its solubility in DMSO is below 25 mg/mL. Recently, solvent compositions of dichloromethane and primary alcohol mixtures with high-solubility telmisartan have been reported [9]. In particular, the solubility of telmisartan in pure methanol and dichloromethane was 2 and 14 mg/g, respectively, whereas, in the range of 0.5–0.9 of dichloromethane mass fractions in dichloromethane and methanol mixtures, it was above 50 mg/g at 25 °C. Thus, dichloromethane and methanol mixtures were applied as a solvent for telmisartan SAS processing.

### 2.4. Experimental Design for Finely Tailoring Nano-Micro-Ranged Particle Sizes

Further study on the SAS process was conducted to control the telmisartan nano-microparticle size in the selected ranges of SAS process conditions based on the results of the screening study described in Section 2.3. The effects of the concentration of the drug solution (X_1_, 20 mg/g and 50 mg/g) and the ratio of dichloromethane in a solvent mixture with methanol (X_2_, 50 and 90) were investigated using FD. The temperature and pressure were fixed at 40 °C and 150 bar, respectively, based on the four-dimensional (4D) plot obtained from the screening study. The injection rates of the drug solution and CO_2_ were reset to 0.75 g/min and 40 g/min, respectively, for process efficiency. The dependent variables for a total of 13 experimental points (3^2^ FD + four center points) were the particle sizes (Y), and experimental design and data analyses were performed using the Design Expert 11.0 software (Stat-Ease, Inc.).

### 2.5. Solid-State Characterization

#### 2.5.1. SEM Analysis

The morphology of pure telmisartan and all samples prepared using the SAS process was observed using SEM (SUPRA 25 or 40; Zeiss, Oberkochen, Germany). To enhance the electrical conductivity of the sample, each sample was fixed on an aluminum stub with a carbon conductive tape and sputter-coated with a gold layer at 20 mA for 100 s by an ion sputter coater. The SEM images were scanned using an acceleration voltage of 5 kV.

#### 2.5.2. Particle Size Measurement

The particle size and size distribution of the samples prepared using the SAS process were measured after adding the sample in double-distilled water, suspending it using a vortex mixer, and sonicating it for 1 min to avoid particle aggregation. Then, the suspension was analyzed using a DLS spectrophotometer (ELSZ-1000; Otsuka Eletronics, Osaka, Japan), and each measurement was repeated three times.

#### 2.5.3. XRD Analysis

XRD patterns were obtained using an XRD system (Xpert 3; Malvern Panalytical, Almelo, The Netherlands) to evaluate the crystal forms of raw materials and prepared samples. Cu-Kα radiation was generated at a current of 30 mA and voltage of 40 kV. The samples were flattened onto the sample holder and then measured in an angle range of 5–60° (2θ) with a step size of 0.005° and scan speed of 0.05°/s.

#### 2.5.4. FT-IR Analysis

An FT-IR spectrometer equipped with an attenuated total reflectance accessory (Spectrum GX; PerkinElmer, Waltham, MA, USA) was used to identify the chemical structure of the raw materials and samples (prepared using the SAS process). The sample was placed on the ZnSe crystal plate, pressed for contact of the sample with the crystal, and then fixed using the force lever. A total of 16 scans were performed in the spectral range of 600–4000 cm^−1^ with a resolution of 4 cm^−1^.

### 2.6. Kinetic Solubility

For kinetic solubility studies, pure telmisartan and samples prepared using the SAS process (40 mg) were added to 250 mL distilled water and then stirred constantly at 150 rpm and 37.0 ± 0.1 °C. At sampling time points of 5, 10, 15, 30, 45, 60, 120, 180, 300, 360, 720, and 1440 min after the start of the test, 1.5 mL aliquots were collected, then immediately filtered through a syringe filter with a 0.05 μm pore size. Filtered samples were diluted to an appropriate concentration for analysis using an HPLC mobile phase, and the drug concentration was analyzed using a Shimadzu HPLC system (Shimadzu, Tokyo, Japan) consisting of a pump (Model 1525), a column oven (CTO-20A), an auto-sampler (SIL-20 AC), and a UV/Vis detector (SPD-20A). The analysis was performed by placing a C18 column (5 μm, 4.6× 150 mm, Agilent Technologies, Santa Clara, CA, USA) in an oven set at 30 °C. The wavelength was 296 nm and the mobile phase consisted of methanol and 51.8 mM ammonium acetate in a 75:25 volume ratio (*v*/*v*) delivered at a flow rate of 1.0 mL/min.

### 2.7. Powder Dissolution Rate

To examine the effect of particle size on dissolution, a dissolution study was performed on the samples prepared using the SAS process with a USP Type-II (Paddle) dissolution apparatus. Samples corresponding to 40 mg were added to 900 mL of the dissolution medium (pH 6.8 phosphate buffer containing 0.5% SLS) rotating at 50 rpm at 37 ± 0.1 °C. Then, 3 mL of the dissolution sample was withdrawn at 5, 10, 15, 30, 45, 60, 90, and 120 min after sample addition. It was filtered through a syringe filter with a 0.05 μm pore size and diluted with methanol. The drug concentration was analyzed under the same conditions as the HPLC conditions used in the kinetic solubility study.

### 2.8. Pharmacokinetic Study in Rats

#### 2.8.1. Method

All animal experiments were approved by the animal ethics committee of Kyungsung University and conducted in accordance with the “Guidelines for Care and Use of Laboratory Animals” of Kyungsung University (No. 19-008A). Male Sprague Dawley rats (7 weeks old; weighing 200–240 g) were purchased from Hyochang Science (Daegu, Republic of Korea) and randomly divided into four groups (*n* = 6). All animals were fasted for approximately 12 h pre-dosing and 2 h post-dosing and had free access to food and water the rest of the time. Each rat was weighed before starting the administration. Pure telmisartan and samples prepared using the SAS process (198, 595, and 1050 nm nano-microparticles) were sufficiently dispersed in distilled water and then orally administered at a dose of 4 mg/kg. Blood samples of approximately 300 μL were collected through the jugular vein at the following time points: 0 (pre-dosing), 0.5, 1, 2, 3, 4, 6, 8, 12, 24, and 48 h after administration. Blood samples were then subsequently centrifuged for 5 min at 10,000 rpm. The obtained plasma samples were transferred into tubes and stored at −70 °C until HPLC analysis.

#### 2.8.2. HPLC Analysis

A plasma sample (100 μL) was spiked with an internal standard solution containing raloxifene at a concentration of 200 ng/mL (100 μL), to which methanol (200 μL) was added. The mixture was vortex-mixed for approximately 5 min, followed by centrifugation at 13,000 rpm for 20 min (20 °C). The supernatant was carefully transferred to a 5 mL glass tube and then maintained at 50 °C under a nitrogen purge using a heating block to remove the solvent. After methanol (100 μL) was added to the dried residue and mixed, HPLC analysis was performed for telmisartan quantification. The same HPLC system used in the kinetic solubility study was applied, and a C18 column (Gemini; 5 μm, 4.6 × 150 mm, Phenomenex, Torrance, CA, USA) was used. The mobile phase consisted of a mixture of methanol and 51.8 mM ammonium acetate in a 70:30 volume ratio (*v*/*v*) and injected at a rate of 1.0 mL/min. The temperature and detection wavelength were set at 30 °C and 296 nm, respectively.

#### 2.8.3. Pharmacokinetic Data Analysis

Pharmacokinetic parameters, such as the maximum plasma concentration of the drug (*C*_max_) and the time to reach the *C*_max_ (*T*_max_), were confirmed using the calculated plasma data, and the area under the concentration–time curve from 0 to 48 h (*AUC*_0→48h_) was computed using the Phoenix WinNonlin software version 8.3 (Certara, Princeton, NJ, USA). In addition, one-way analysis of variance (ANOVA) and Tukey–HSD multiple comparisons test were performed to identify the significant differences in the particles prepared according to the process parameters.

## 3. Results and Discussion

### 3.1. Effect of SAS Process Parameters on the Particle Formation of Telmisartan

**Figure 2:**
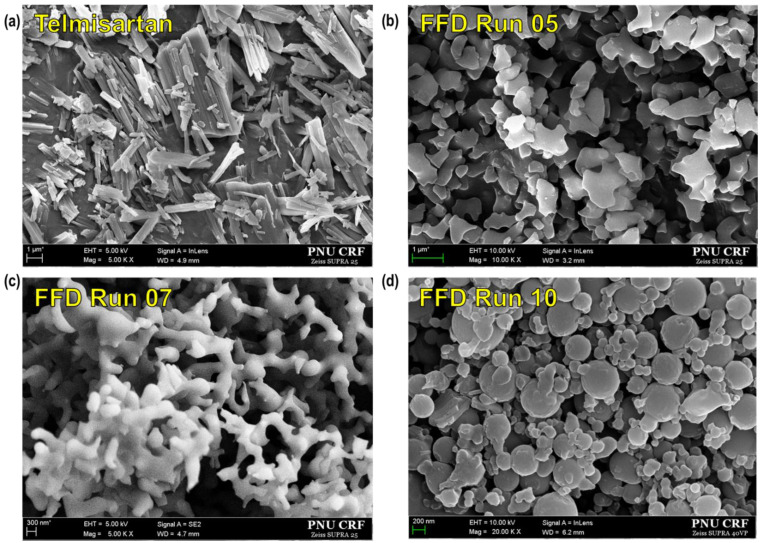
SEM images of telmisartan amorphous particles obtained using the SAS process: (**a**) unprocessed telmisartan; SAS-processed telmisartan corresponding to (**b**) Run 05, (**c**) Run 07, and (**d**) Run 10 of the fractional factorial design (FFD) matrix.

Telmisartan (C_33_H_30_N_4_O_2_; Form A) was obtained by Dong-A ST Co., Ltd. (Seoul, Republic of Korea). The effect of SAS process parameters, such as the solvent mixture ratio of dichloromethane to methanol, drug solution concentration, temperature, pressure, drug solution injection rate, and CO_2_ flow rate, on the formation of telmisartan particles was investigated in the screening study. All experiments were carried out according to the design matrix generated from a 2^6−2^ FFD with three center points. The particle size and crystal form of the telmisartan particles prepared using the SAS process are presented in Table 1. The unprocessed telmisartan had an average particle size of 2 μm and appeared as a rod-like structure with an irregular length (Figure 2a). Conversely, the processed telmisartan obtained using the SAS process had a particle size that ranged from 362 to 1900 nm, which was relatively small compared to that of unprocessed telmisartan. In addition, a tendency for the particles to appear more spherical was observed as the ratio of the solvent mixture was decreased (Figure 2b–d), even though it should be noted that the changes in other process variables were combined with the solvent ratio parameter. This tendency was similarly observed in experimental results based on FD, where other process variables were the same and only the solvent ratio was changed. This is discussed again in Section 3.2 below.

**Figure 3:**
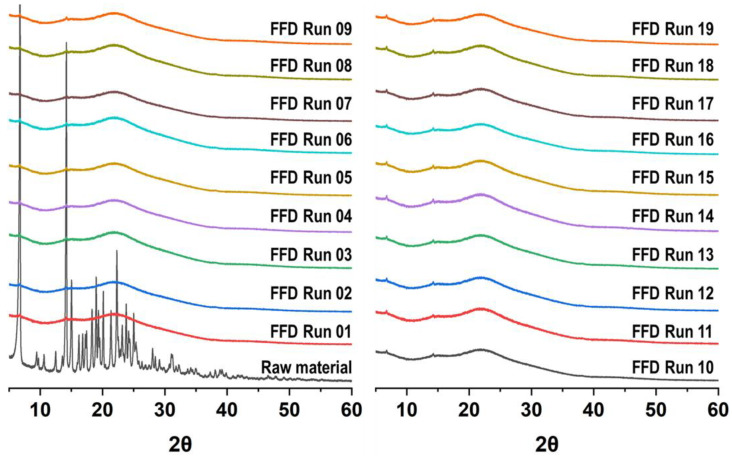
XRD patterns of telmisartan before and after the SAS process.

The results of the XRD analysis, which was performed to investigate the crystal form of the unprocessed and processed particles, are shown in Figure 3. As reported in the literature [4,10], unprocessed telmisartan had characteristic peaks at diffraction angles of 6.8°, 14.2°, and 22.3° in the polymorphic form A. Particles processed using the SAS process did not have any other noticeable diffraction peaks compared to unprocessed telmisartan, indicating that telmisartan exists in an amorphous form.

**Figure 4:**
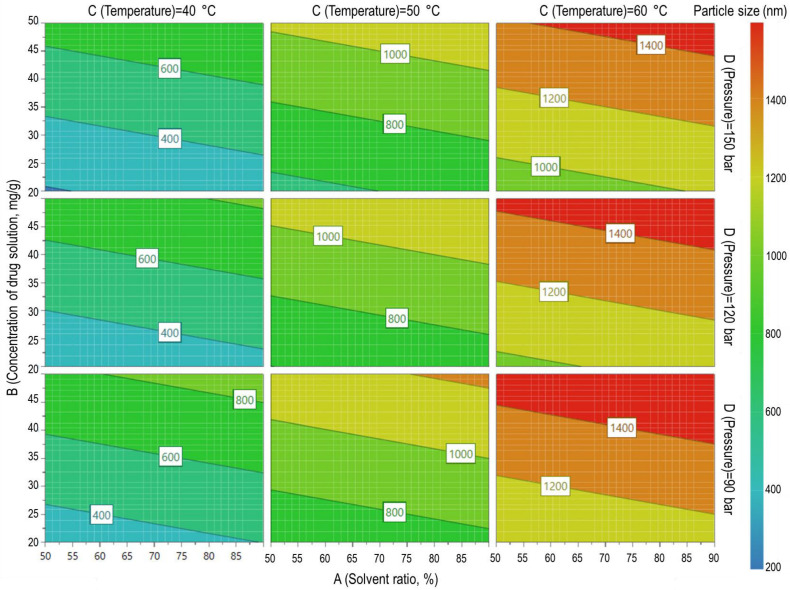
Response 4D contour plot obtained through regression analysis.

ANOVA was carried out to identify the parameters of the SAS process that affect particle preparation, and a 4D contour plot was obtained based on these results, which are shown in Table 2 and Figure 4, respectively. The analysis performed was considered appropriate because the *p*-values for the generated model were < 0.05, the value of lack-of-fit was not significant, and the R^2^ value for the model was 0.9615, which was > 0.8. Four independent variables, the solvent mixture ratio (*p*-value < 0.1), drug solution concentration (*p*-value < 0.05), temperature (*p*-value < 0.05), and pressure (*p*-value < 0.1), were considered mainly to analyze their effects on the particle size of SAS-processed telmisartan within the set range. The process parameters with a *p*-value > 0.1 were excluded from the discussion for particle size. Among them, the drug solution concentration and temperature, with *p*-values < 0.05, appeared to have the greatest influence on particle size. These particle size alterations caused by the variation in SAS process parameters can be explained by the fact that the size and shape of particles prepared using the SAS process are mainly determined by two mechanisms: (i) the evaporation of the solvent into the SC-CO_2_ phase and (ii) the extraction of the solvent via diffusion of the SC-CO_2_ into the droplets [20,21]. The notable results of changes in particle formation characteristics induced by variation in the process parameters of SAS are as follows.

The increase in temperature in the SAS process from 40 °C to 60 °C was found to have the greatest influence on the particle size reduction in SAS-processed telmisartan. It was also found that the effect of an increase in particle size is greater in mixed solvents with a high proportion of highly volatile dichloromethane. It is generally understood that particle formation occurs primarily through excessive solvent evaporation in the SAS process, leading to larger particles [21,22,23,24,25]. In addition, the solubility of telmisartan in the solvent mixtures was decreased by lowering the ratio of dichloromethane from 90 to 50 [26]. These differences in solubility could affect nucleation and crystal growth following supersaturation during the SAS process, thereby varying the characteristics of particle formation [7,27]. Thus, it can be suggested that the particle size of telmisartan can be reduced as the temperature and fraction of volatile solvent decrease in the SAS process.

The increase in the drug solution concentration resulted in a particle size increase. As the concentration of the drug solution decreases to 20 mg/g, the saturation and precipitation of the drug become very slow during the droplet expansion step, forming smaller particles, thus resulting in a decrease in particle size. However, above a certain concentration, larger particles are prepared because the particle growth dominates the nucleation step [21,22,27]. Additionally, in the SAS process, an increase in the drug solution concentration increases the viscosity of the solution, which increases the size of the drug solution droplets generated by spraying into the SC-CO_2_. This increases the resistance to the diffusion in and out of the SC-CO_2_ into the drug solution droplets, resulting in a decrease in the solvent extraction efficiency [26,27,28,29,30,31,32]. Meanwhile, an increase in the pressure of the SAS process from 90 bar to 150 bar can result in a particle size decrease. This is because the volume expansion of the liquid phase increases the mass transfer between the drug and CO_2_, and the nucleation rate. As a result, the drug molecules are precipitated in a more compact form and the particle size tends to be slightly smaller. These results suggest that particle size reduction can be achieved by efficient solvent extraction using SC-CO_2_ through an increase in the CO_2_ density at higher pressure and/or a decrease in the drug solution concentration. Faster flow rates of the drug solution and CO_2_ tended to decrease particle size; however, their effects were statistically negligible compared to the greater influence of other independent variables.

Such findings suggest that the particle size and morphology of SAS-processed telmisartan were mainly controlled by the solvent mixture ratio and the concentration of the drug solution [4,10]. Thus, the ratios of the drug solution concentration and solvent mixture were adjusted in further investigations to confirm the region in which nano-microparticles with a size < 1.5 μm can be obtained. For these experiments, the SAS process temperature and pressure were fixed to 40 °C and 150 bar, respectively, which were deemed suitable for preparing nano-microparticles with different sizes, and the flow rates of the drug solution and SC-CO_2_ were set to the maximum to achieve process efficiency.

### 3.2. Fine-Tailoring Telmisartan Particle Size

**Figure 5:**
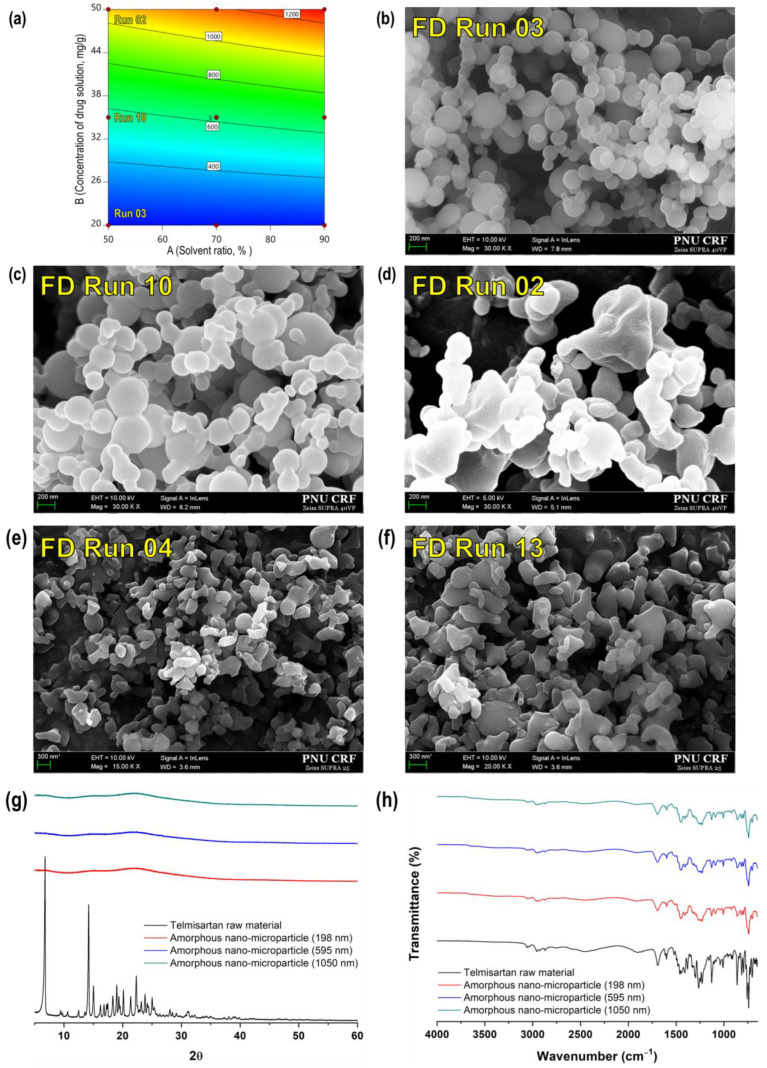
(**a**) Contour plot for particle size; SEM images: (**b**) Run 3 sample (198 nm), (**c**) Run 10 sample (595 nm), (**d**) Run 02 sample (1050 nm), (**e**) Run 4 sample, (**f**) Run 13 sample, (**g**) XRD patterns, and (**h**) FT-IR spectrum of telmisartan particles prepared according to the factorial design (FD) matrix using the SAS process.

Further experiments were performed to determine the conditions for preparing amorphous nano-microparticles of telmisartan with various particle sizes within a range < 1.5 μm by controlling the solvent mixture ratio and the drug solution concentration. Amorphous nano-microparticles were prepared according to the experimental design matrix derived using the FD; on the basis of the results of the screening study, the temperature, pressure, drug solution injection rate, and CO_2_ flow rate were set to 40 °C, 150 bar, 0.75 g/min, and 40 g/min, respectively. Amorphous nanoparticles of telmisartan with various particle sizes were prepared, as shown in Table 3 and Figure 5b–d. Both the solvent ratio and the drug solution concentration had significant effects on particle size—the particle size decreased as the two variables decreased. The smallest particles, measuring 198 nm, were prepared using a 50% solvent ratio and a 20 mg/g drug solution concentration, and the largest particles, measuring 1290 nm, were prepared using a 90% solvent ratio and a 50 mg/g drug solution concentration.

The results of the ANOVA analysis and contour plot are shown in Table 4 and Figure 5a. The *p*-value of the model was < 0.05, the value of lack of fit was not significant, and the R^2^ value was > 0.8. The model was considered to be suitable based on these findings; the two variables affected the size of the particles prepared using the SAS process according to the statistical analysis (*p*-value < 0.05), similar to the experimental results.

In this experiment, it was further confirmed that the particles appeared more spherical as the solvent ratio of dichloromethane in a mixture with methanol decreased in the range of 50–90%, as described in Section 3.1 above. The same trend was observed in Runs 3, 4, and 13 (Figure 5b,e,f), where other SAS process parameters were the same and only the solvent ratio was changed by the FD.

The lower the ratio of dichloromethane in the solvent mixture, the higher the probability of preparing spherical or nearly spherical particles (Figure 5b–d). It was confirmed from the XRD pattern that the particles produced through the SAS process were amorphous (Figure 5g) [11,12]. The FT-IR spectra of unprocessed telmisartan and SAS-processed telmisartan are shown in Figure 5h. The FT-IR spectrum of unprocessed telmisartan had the following characteristic peaks: 3063 cm^−1^ (aromatic C-H stretch), 2957 cm^−1^ (aliphatic C-H stretch), 1697 cm^−1^ (carbonyl stretch), 1599 cm^−1^ (aromatic C=C bend and stretch), 1381 cm^−1^ (CH_3_ bending vibrations), 1350–1000 cm^−1^ (C-N stretching vibrations), and 740 and 757 cm^−1^ (ring vibration due to 1, 2-disubstituted benzene). These bands were more clearly observed and sharper than those of the amorphous form (SAS-processed telmisartan). Moreover, there were significant differences between the FT-IR spectrum of processed and unprocessed telmisartan and a significant difference in the 1350–640 cm^−1^ region when telmisartan changed from a crystalline to an amorphous form [11,12]. Therefore, in the SAS process, telmisartan with various particle sizes within the submicron range could be obtained by changing the concentration of the drug solution in the range of 20–50 mg/g at a process condition of 40 °C and 150 bar, with a 0.75 g/min injection rate of drug solution and 40 g/min CO_2_ flow rate.

### 3.3. Effect of Particle Size on Dissolution and Oral Bioavailability

For kinetic solubility studies, pure telmisartan and samples prepared using the SAS process (40 mg) had a pH-dependent solubility profile with extremely low solubility in the neutral pH range, which affects small intestine absorption after oral administration, limiting its therapeutic use [33,34,35]. Generally, formulating these drugs into amorphous particles can help improve their solubility and bioavailability [36,37,38]. Thus, the effect of particle size on the kinetic solubility, powder dissolution, and pharmacokinetics of the amorphous telmisartan was evaluated using three different particle sizes (198, 595, and 1050 nm) prepared using the SAS process.

**Figure 6:**
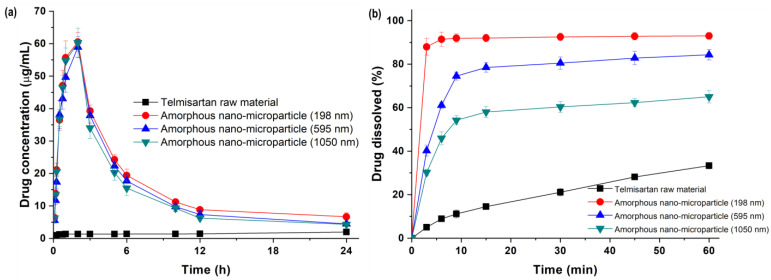
(**a**) Kinetic solubility profile and (**b**) dissolution profiles of unprocessed and SAS-processed telmisartan amorphous nano-microparticles.

The pattern of the supersaturated dissolution of unprocessed telmisartan and SAS-processed telmisartan is shown in Figure 6a. The unprocessed telmisartan reached its highest solubility after 24 h (~60 μg/mL). In contrast, the SAS-processed amorphous telmisartan nano-microparticles showed the maximum supersaturated concentration after 2 h, which then gradually decreased to attain almost the same concentration as the unprocessed telmisartan. This behavior of the processed particles was due to the conversion from an amorphous to a crystalline state.

The dissolution profiles of unprocessed telmisartan and SAS-processed telmisartan are shown in Figure 6b. Only 33.3 ± 2.1% of the unprocessed crystalline telmisartan dissolved within 60 min and continued to increase until the end of the test. The amorphous telmisartan nanoparticles prepared using the SAS process showed increased dissolution rates compared to unprocessed telmisartan and reached dissolution equilibrium within 10 min, and differences were found according to the particle size. The degree of dissolution after 60 min for the nano-microparticles with a particle size of 198 nm was 2.8-fold (93.0 ± 1.6%) higher, and the dissolution rates of the nano-microparticles with particle sizes of 595 nm and 1050 nm were 2.6-fold (86.3 ± 2.7%) and 2.0-fold (67.0 ± 3.5%) higher, respectively, compared to unprocessed telmisartan. These results suggest that telmisartan particles prepared in the amorphous form using the SAS process have excessive free energy, which increases thermodynamic instability and improves solubility [4,11,38]. It also means that surface area increases with a smaller particle size, leading to a shorter diffusional distance and, subsequently, a higher dissolution rate [39,40].

The results of powder dissolution were also applied to the Hixson–Crowell kinetic model to explain the drug release profile of telmisartan particles. The estimated dissolution rate (*k*) and simulated 50% dissolution time (*t*_50%_) of telmisartan using the Hixson–Crowell model are shown in Table 5. As the particle size of SAS-processed telmisartan decreased, the dissolution rate increased and the *t*_50%_ decreased; unprocessed telmisartan showed the smallest dissolution rate and the largest value of *t*_50%_.

**Figure 7:**
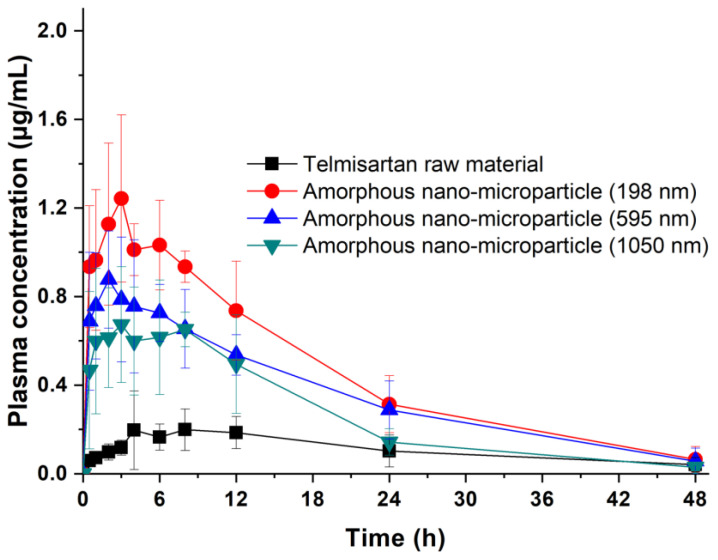
Plasma concentration-time profiles of telmisartan in rats after oral administration of telmisartan amorphous nano-microparticles. Data are expressed as the mean ± standard deviation (*n* = 6).

Comparing the biopharmaceutical characteristics between unprocessed telmisartan and SAS-processed telmisartan submicron particles with three different particle sizes after a single oral administration, in vivo pharmacokinetic data and the plasma concentration versus time profile are shown in Table 6 and Figure 7, respectively. The unprocessed telmisartan had a *C*_max_ of ~0.3 ± 0.2 μg/mL at a *T*_max_ of 5.8 ± 2.0 h after oral administration, with an *AUC*_0→48h_ of 5.3 ± 2.1 μg∙h/mL. In contrast, the *C*_max_ of SAS-processed telmisartan was 1.4 ± 0.3 μg/mL at 2.6 ± 1.2 h for a 198 nm particle, 1.0 ± 0.2 μg/mL at 3.7 ± 2.0 h for a 595 nm particle, and 0.9 ± 0.2 μg/mL at 3.2 ± 2.6 h for a 1050 nm particle, with *AUC*_0→48h_ values of 22.9 ± 2.9 μg∙h/mL, 17.1 ± 2.7 μg∙h/mL, and 12.8 ± 3.7 μg∙h/mL, respectively. Both *C*_max_ and *AUC*_0→48h_ indicated significant differences between SAS-processed and unprocessed telmisartan, with increases in the following order (*p* < 0.05): 198 nm nano-microparticle > 595 nm nano-microparticle > 1050 nm nano-microparticle > unprocessed telmisartan. The oral bioavailability of SAS-processed telmisartan with a particle size of 198 nm was 4.3-fold higher than that of the unprocessed telmisartan. Thus, it was suggested that the surface area of telmisartan effectively increased as the particle size decreased in the SAS process, causing an increase in the dissolution rate.

**Figure 8:**
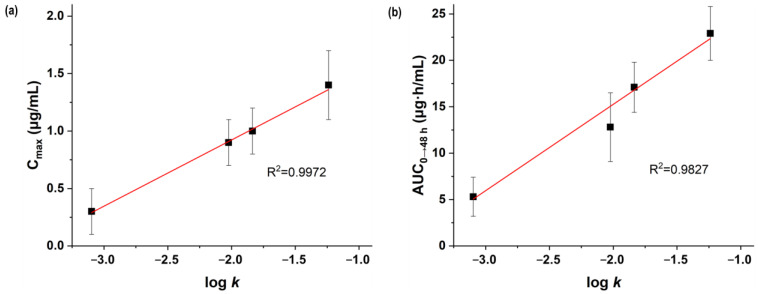
Correlation between the in vitro dissolution rate and in vivo pharmacokinetic data of unprocessed and SAS-processed telmisartan particles: (**a**) in vitro *k* (dissolution rate, mg^1/3^/min) vs. in vivo *C*_max_; (**b**) in vitro *k* (dissolution rate, mg^1/3^/min) vs. in vivo *AUC*_0→48h_ (the *x*-axis has a logarithmic scale).

As the *C*_max_ and *AUC*_0→48h_ of the processed telmisartan increased with a decrease in particle size, it was evident that the oral absorption of telmisartan could be controlled by the particle size. Additionally, the dissolution rate and pharmacokinetic parameters (*AUC*_0→48h_ and *C*_max_) were plotted to evaluate their correlation (Figure 8). The R^2^ for both plots was > 0.8, and the *C*_max_ values showed a relatively high correlation compared to the *AUC*_0→48h_ values; therefore, in vivo pharmacokinetic parameters could be predicted using the in vitro dissolution rate of telmisartan particles. The in vitro and in vivo data showed that even when the particles were in the amorphous form, there existed a correlation according to the particle size. This explanation is supported by the published literature on the improved biopharmaceutical properties of telmisartan due to a particle size reduction. Bajaj et al. reported that an 82.63 nm nanosuspension showed a 15.7 times higher *C*_max_ and a 10.6 times higher *AUC*_0→48h_ than a 2.5 μm microsuspension for oral administration in Wistar rats [13]. Enose et al. showed that amorphous microparticles (approximately 2 μm in SEM image) had a 13.3-fold increase in *C*_max_ and a 5.3-fold increase in *AUC*_0→48h_ compared to crystalline acicular microparticles (approximately 5 μm in SEM image) for oral pharmacokinetic study in Sprague Dawley rats [15]. In addition, Patel et al. reported that aqueous dispersions of tablets containing 341 nm nanoparticles showed a 1.5-fold increase in *C*_max_ and a 1.4-fold increase in *AUC*_0→48h_ compared to aqueous dispersions of marketed tablets prepared with coarse microparticles when orally administered to Wistar rats [14]. Therefore, it can be confirmed that preparing amorphous nano-microparticles for poorly water-soluble drugs is an effective strategy to improve their dissolution and bioavailability.

## 4. Conclusions

In this study, amorphous telmisartan nano-microparticles with particle sizes ranging from 198 to 1201 nm were prepared using the SAS process and a mixture of dichloromethane and methanol. According to the screening experiments, particle size was mainly controlled by temperature and drug solution concentration. Moreover, the size and morphology of particles were demonstrated to be tailored by the drug solution concentration and solvent mixture ratio at a fixed temperature along with the pressure conditions of the precipitation vessel. Compared to the crystalline microparticles, the amorphous nano-microparticles had higher solubility and a rapid dissolution rate. The dissolution rate and oral absorption of telmisartan were found to increase as the particle size of the amorphous nano-microparticles decreased. These results confirm that the size and morphology of drug particles can be efficiently controlled by the solvent compositions (ratio) of dichloromethane and methanol. Consequently, it was shown that acceptable ranges of process conditions for obtaining nano-microparticles using the SAS process with mixed solvents can be found through efficient control of the solvent composition based on the experimental design method for drugs with limitations in the application of SAS when using a single solvent. In addition, this study demonstrated that the preparation of amorphous telmisartan nano-microparticles in this method could be an effective approach to enhance the solubility, dissolution, and oral absorption of the poorly water-soluble telmisartan. Therefore, it can be suggested that the use of a mixed solvent system in SAS processes and control of their ratio in combination with other process variables can be utilized to further expand the opportunities for developing solid nano-microparticle formulations with enhanced bioavailability for poorly water-soluble drugs.

## Figures and Tables

**Table 1:** Fractional factorial design matrix of variables and the obtained response.

	Factors						Response	
	X_1_	X_2_	X_3_	X_4_	X_5_	X_6_	Y_1_	Y_2_
	A: Solvent Ratio (%, *w*/*w*)	B: Concentration of Drug Solution (mg/g)	C: Temperature (°C)	D: Pressure (bar)	E: Injection Rate of Drug Solution (g/min)	F: CO_2_ Flow Rate (g/min)	Particle Size (nm)	Crystal Form
1	50	50	60	90	0.25	20	1555	Amorphous
2	50	50	60	150	0.25	40	1295	Amorphous
3	70	35	50	120	0.5	30	627	Amorphous
4	90	50	60	150	0.75	40	1628	Amorphous
5	50	20	60	150	0.75	20	952	Amorphous
6	90	50	60	90	0.75	20	1900	Amorphous
7	70	35	50	120	0.5	30	552	Amorphous
8	50	50	40	150	0.75	20	738	Amorphous
9	90	20	60	90	0.25	40	1072	Amorphous
10	50	20	40	150	0.25	40	362	Amorphous
11	50	20	40	90	0.25	20	405	Amorphous
12	90	20	60	150	0.25	20	1000	Amorphous
13	90	50	40	150	0.25	20	769	Amorphous
14	90	50	40	90	0.25	40	805	Amorphous
15	50	20	60	90	0.75	40	1026	Amorphous
16	70	35	50	120	0.5	30	672	Amorphous
17	90	20	40	150	0.75	40	393	Amorphous
18	50	50	40	90	0.75	40	783	Amorphous
19	90	20	40	90	0.75	20	432	Amorphous

**Table 2:** Regression analysis for particle size in fractional factorial design.

Source		Sum of Squares	df	Mean Square	F-Value	*p*-Value	
Model		3.101 × 10^6^	6	5.169 × 10^5^	45.83	<0.0001	significant
A-Solvent ratio		48,730.56	1	48,730.56	4.32	0.0618	
B-Concentration of drug solution		9.173 × 10^5^	1	9.173 × 10^5^	81.34	<0.0001	
C-Temperature		2.060 × 10^6^	1	2.060 × 10^6^	182.66	<0.0001	
D-Pressure		44,205.06	1	44,205.06	3.92	0.0733	
E-Injection rate of drug solution		21,682.56	1	21,682.56	1.92	0.1930	
F-CO_2_ flow rate		9360.56	1	9360.56	0.8300	0.3818	
Curvature		2.713 × 10^5^	1	2.713 × 10^5^	24.05	0.0005	
Residual		1.241 × 10^5^	11	11,277.37			
Lack of Fit		1.167 × 10^5^	9	12,966.78	3.53	0.2403	not significant
Pure Error		7350.00	2	3675.00			
Cor Total		3.497 × 10^6^	18				
Std. Dev	106.19	R-squared	0.9615		Pred R-squared		0.8805
Mean	892.95	Adj R-squared	0.9406		Adeq R-squared		22.2614
C.V.%	11.89						

**Table 3:** Regression analysis for particle size in factorial design.

Run	Factors		Response
	X_1_	X_2_	Y
	A: Solvent Ratio (%, *w*/*w*)	B: Concentration of Drug Solution (mg/g)	Particle Size (nm)
1	70	35	610
2	50	50	1050
3	50	20	198
4	70	20	210
5	90	50	1290
6	70	35	625
7	70	50	1200
8	70	35	601
9	70	35	639
10	50	35	595
11	90	35	650
12	70	35	617
13	90	20	235

**Table 4:** Regression analysis for particle size in factorial design.

Source		Sum of Squares	df	Mean Square	F-Value	*p*-Value	
Model		1.447 × 10^6^	4	3.617 × 10^5^	757.64	<0.0001	significant
A-Solvent ratio		18,370.67	1	18,370.67	38.48	0.0003	
B-Concentration of drug solution		1.399 × 10^6^	1	1.399 × 10^6^	2929.77	<0.0001	
AB		10,302.25	1	10,302.25	21.58	0.0017	
B^2^		19,452.53	1	19,452.53	40.74	0.0002	
Residual		3819.46	8	477.43			
Lack of Fit		2976.26	4	744.07	3.53	0.1247	not significant
Pure Error		843.20	4	210.80			
Cor Total		1.451 × 10^6^	12				
Std. Dev	21.85	R-squared	0.9974		Pred R-squared		0.9854
Mean	655.38	Adj R-squared	0.9961		Adeq R-squared		79.4287
C.V.%	3.33						

**Table 5:** Dissolution rate and simulated 50% dissolution time for telmisartan raw material and amorphous nano-microparticles determined using the Hixson–Crowell equation.

Formulation	Dissolution Rate, *k* (mg^1/3^/min)	Simulated 50% Dissolution Time, *t*_50%_ (min)
Telmisartan raw material	0.0008	88.2
Amorphous nano-microparticle (198 nm)	0.0577	1.2
Amorphous nano-microparticle (595 nm)	0.0146	4.8
Amorphous nano-microparticle (1050 nm)	0.0095	7.4

Note: Hixson–Crowell model: *M*_0_^1/3^ − *M*_t_^1/3^ = *kt*, where *M*_0_ is the initial mass of telmisartan, *M*_t_ is the mass of telmisartan released at time (*t*), and *k* is the dissolution rate constant.

**Table 6:** In vivo pharmacokinetic data for telmisartan amorphous nano-microparticles.

Formulation	In Vivo Pharmacokinetic Data		
	*AUC*_0→48h_ (μg·h/mL)	*C*_max_ (μg/mL)	*T*_max_ (h)
Telmisartan raw material	5.3 ± 2.1	0.3 ± 0.2	5.8 ± 2.0
Amorphous nano-microparticle (198 nm)	22.9 ± 2.9	1.4 ± 0.3	2.6 ± 1.2
Amorphous nano-microparticle (595 nm)	17.1 ± 2.7	1.0 ± 0.2	3.7 ± 2.0
Amorphous nano-microparticle (1050 nm)	12.8 ± 3.7	0.9 ± 0.2	3.2 ± 2.6

## Data Availability

Data are contained within the article.
